# Barriers and facilitators to COVID-19 testing among staff and parents from San Diego schools

**DOI:** 10.1186/s12889-023-15854-x

**Published:** 2023-06-05

**Authors:** Megan Nguyen, Marlene Flores, Anh Van Vo, Vinton Omaleki, Samantha Streuli, Rebecca Fielding-Miller

**Affiliations:** 1 San Diego Herbert Wertheim School of Public Health, University of California, San Diego, USA; 2 Johns Hopkins Bloomberg School of Public Health, Baltimore, USA

**Keywords:** Covid-19, Testing, Schools, Barriers, Facilitators, Health disparities, Children’s health

## Abstract

COVID-19 testing is an important risk mitigation strategy for COVID-19 prevention in school settings, where the virus continues to pose a public health challenge for in-person learning. Socially vulnerable school communities with the highest proportion of low-income, minority, and non-English speaking families have the least testing access despite shouldering a disproportionate burden of COVID-19 morbidity and mortality. Through the Safer at School Early Alert (SASEA) program, we investigated community perceptions of testing in San Diego County schools, with a focus on barriers and facilitators from the perspective of socially vulnerable parents and school staff. Using a mixed-methods approach, we administered a community survey and conducted focus group discussions (FGDs) with staff and parents from SASEA-affiliated schools and childcares. We recruited 299 survey respondents and 42 FGD participants. Protecting one’s family (96.6%) and protecting one’s community (96.6%) were marked as key motivators to testing uptake. School staff in particular reported that the reassurance of a negative status mitigated concerns about COVID-19 infection in schools. Participants expressed that COVID-19-related stigma, loss of income as a result of isolation/quarantine requirements, and lack of multilingual materials were the most significant barriers to testing. Our findings suggest that the testing barriers faced by school community members are predominantly structural. Testing uptake efforts must provide support and resources to manage the social and financial consequences of testing while continuously communicating its benefits. There is a clear need to continue to incorporate testing as a strategy to maintain school safety and facilitate access for vulnerable community members.

## Background

COVID-19 testing is an important risk mitigation strategy for COVID-19 prevention in school settings [1] where the virus continues to pose a dynamic public health challenge [2]. Along with masking and community vaccination [3–5] school testing is a key way to mitigate spread during in-person learning [6, 7]. Academic discourse on testing in schools has focused on attitudes towards testing [8], the effectiveness of testing for optimal school operations [9, 10], and barriers to testing [11, 12].

Socially vulnerable school communities with the highest proportion of low-income, minority, and non-English speaking families [13] also have the least testing access despite shouldering a disproportionate burden of COVID-19 morbidity and mortality [14, 15]. This is compounded by the structural drivers - such as overcrowded housing and precarious forms of employment - that continue to place vulnerable populations at heightened risk of COVID-19 infection [16].

While antigen tests taken at-home have dramatically increased testing accessibility, they come with their own issues of uptake, supply, and cost [17]. Even when tests are available, testing is still not always equitable. Testing uptake among socially vulnerable communities is lower due to factors such as lack of sick leave, limited testing locations, and a historical mistrust in formal health systems and research [18]. Although testing can be valuable, there exists hesitancy towards using this tool. It is necessary to understand barriers to testing as it remains an important COVID-19 mitigation strategy which can reduce health disparities for vulnerable populations.

We conducted a mixed-methods study to understand community perceptions of testing in San Diego County schools, with a focus on barriers and facilitators from the perspective of socially vulnerable parents and school staff.

## Methods

### Study design

We utilized a mixed-methods approach that combined virtual focus group discussions (FGDs) with a self-administered online cross-sectional survey. Recruitment and data collection occurred between December 2020 and March 2021, before vaccines were available to any children. The research team, composed of undergraduate students, graduate-level students, and research staff and faculty from the University of California San Diego Herbert Wertheim School of Public Health, received training on best practices for conducting qualitative research with participants from diverse and under-served populations.

### Setting

San Diego is a diverse county with nearly one fifth of residents being foreign-born and a large immigrant and refugee population [19]. In late 2020, 15 school sites were partnered with the Safer at School Early Alert (SASEA) pilot, a COVID-19 surveillance and routine diagnostic testing program at schools and childcare sites in select communities across San Diego County. These schools were selected due to elevated cases of COVID-19 per 1,000 residents and were located in census tracts with high social vulnerability according to the CDC Social Vulnerability Index (SVI) scores [20]. School eligibility parameters were set by the County of San Diego to ensure that participating schools were considered socially vulnerable. Schools contacted were not randomly selected by the County of San Diego or the University of California, San Diego. Principals of eligible schools were able to opt-in to the SASEA program. All 15 enrolled sites offered some form of hybrid (in-person and online) learning at the time the data were collected, which impacted parental choice to keep their child(ren) at home or send them to school in-person.

### Participants

Participants were eligible for this study if they identified themselves as a staff member and/or a parent or guardian of a student at one of the 15 participating sites. Through convenience sampling, we recruited staff members and parents or guardians using two different paper flyers distributed in-person at schools and childcare centers, as well as two rounds of email communications, one to recruit for FGDs and one to recruit for the online surveys. Recruitment information was first provided by the research team to school principals and administrators to then send out directly to all school parents via email. Recruitment continued until the research team determined that thematic saturation had been reached. FGD participants received $25 Visa gift cards for their time. All eligible persons were encouraged to complete the online survey after entering their name into a randomized raffle to win 1 of 3 $250 Visa gift cards, although there was no direct compensation for survey participation. This study was approved by the University of California, San Diego Institutional Review Board, with protocol number 201,627. Preliminary and de-identified results were shared with schools in follow up presentations.

All focus group discussions (FGDs) were conducted over Zoom by qualitative researchers [MN, MF, AM, AV, DD, TL, VO] in English or Spanish using a semi-structured field guide. FGD domains included participant attitudes and perspectives on COVID-19 diagnostic testing and risk mitigation efforts within school communities. All FGDs were digitally recorded, transcribed verbatim, and translated into English as needed.

A link for a self-administered online survey was distributed to all parents and staff affiliated with one of the 15 elementary school and childcare sites enrolled in the SASEA project. The online survey assessed community attitudes towards COVID-19, masking, contact tracing, testing, isolation, and quarantine using primarily multiple-choice questions, including “other” options to allow participants to provide short open-ended responses if their answers are not covered by the available multiple choice options. This survey was available in both English and Spanish. The Spanish version was created by two native Spanish-speaking members of the research team who translated and cross reviewed for accuracy.

### Data analysis

We used an exploratory mixed methods approach to data analysis, as this had the best approach to capturing individual attitudes towards testing while ensuring statistical relevance [21]. Preliminary qualitative analyses focused on identifying predominant themes across transcripts. These themes informed the community survey design. Qualitative analysis used Creswell’s iterative data analysis spiral [22]. No predetermined theory or lens was selected at the beginning of study; rather a grounded theory process was chosen as the best choice for determining how parents and staff felt about testing. After the first round of data collection, the study team developed a codebook to identify emergent themes related to COVID-19 diagnostic testing. We then conducted basic descriptive univariate statistics of the community survey data pertaining to testing behaviors and attitudes to better understand the prevalence of themes that emerged from focus group discussions. These statistics were used to triangulate our discussion of saturation, rather than to indicate generalizability across all school communities.

### Potential biases

Due to virtual modes of data collection and recruitment, participants without internet access were inherently excluded from our sample. Although this may introduce systematic bias in our reporting, we believe the data collected still captured a wide range of attitudes and experiences regarding testing.

## Results

We conducted 15 focus group discussions (FGDs) with 42 participants. 13 FGDs were conducted in English and 2 were conducted in Spanish. 8 FGDs were conducted with school staff members and 7 were conducted with parents or guardians. Two-hundred and ninety-nine individuals participated in the online survey. Of these, 135 (45.9%) identified as a parent or guardian, 140 (47.6%) identified as a staff member, and 19 (6.5%) identified as both. A majority of our respondents identified as female (85.8%), just under two-thirds were white (64.2%), and just over half identified as Hispanic/Latino (52.9%). 232 (92.1%) participants had health insurance, and a vast majority of participants had a high school diploma or higher (95.9%). 20 individuals (6.7%) took the survey in Spanish (Table 1). In the FGDs and surveys, participants were asked to elaborate on barriers and facilitators to testing.

**Table 1:** Demographics Table (N = 299)

*Mean Age* (n = 248)	39.85
*Gender* (n = 253)	
Female	217 (85.77%)
Male	31 (12.25%)
Declined to Answer	5 (1.98%)
*Race* (n = 257)	
Black or African American	8 (3.11%)
Native American or American Indian	9 (3.50%)
Asian American	10 (3.89%)
Native Hawaiian or other Pacific Islander	6 (2.33%)
White	165 (64.20%)
Other	59 (22.96%)
*Ethnicity* (n = 244)	
Hispanic or Latino	129 (52.87%)
Not Hispanic or Latino	115 (47.13%)
*Highest Level of Education Completed* (n = 249)	
Less than High School	5 (2.01%)
Some High School	5 (2.01%)
High School or Equivalent (ex. GED)	81 (32.53%)
Bachelor Degree	75 (30.12%)
Graduate Degree	83 (33.33%)
*Health Insurance* (n = 252)	
Yes	232 (92.06%)
No	16 (6.35%)
Not Sure	4 (1.59%)1.
*School Role* (n = 294)	
Parent/Guardian	135 (45.92%)
School Staff Member	140 (47.62%)
Both	19 (6.46%)

### Social stigma

While participants overwhelmingly felt that they would not hesitate to share a positive test result with people they had been in close contact with, they expressed that the stigma associated with testing positive within their communities was a barrier to testing. This stigma arose due to the necessity to disclose to others that they have been exposed and should get tested, re-arrange work schedules, and prepare for isolation.

I think just making sure people understand that [a positive COVID status]… it’s not going to be used against them...The issue that I’ve heard come up with a parent before is...they didn’t want them labeled or whatever... if they’re not showing symptoms, they don’t want ‘em kicked out of school because they’ve gotten a positive thing when they could have gone on with life technically. -School Staff

### Loss of income

Many participants reported being wary of testing because they cannot afford to isolate or quarantine. The financial implications of isolation include loss of income from being out of work for several days and corresponding childcare costs.

I think to stay at home, for an emergency, right? It does affect you economically, because we live day to day. But it is something essential that we must do. That [economic pressure] stays in the background. -Parent

When interviewing staff members, school employees noted that in these low-income communities, family members may be considered essential workers so work from home options are not available. Therefore, those who cannot afford to quarantine or isolate due to the financial hardship will then opt out of testing.

### Language

Participants mentioned language as a barrier to testing and noticed that testing sites throughout the community did not have multilingual speakers or resources to accommodate non-English speaking families. As many of our participating school communities have notably large Hispanic/Latino populations in particular, participants expressed concerns about how individuals with limited proficiency in English may struggle with understanding testing protocols.

### Family and community health

Knowledge is power. I like to know this. I want to be careful. I want to protect my family and my elderly and all of that. So it’s like those two schools of thought... the one the people that are like “Ehh I don’t think it’s a thing. I don’t care, if we get it, we get it” versus people who are more conscious of how it’s affecting our community. -Parent

Positive family health was identified as the most prominent facilitator of COVID-19 diagnostic testing. 96.6% of survey respondents (n = 268) marked their family’s well being as an important reason to get tested for COVID-19. FGD participants also overwhelmingly felt compelled to get tested for COVID-19 in order to ensure not just their own safety, but the safety of their families.

Community wellbeing was also identified by survey respondents as a key motivator for COVID-19 diagnostic testing. 96.6% of survey respondents (n = 267) had also marked their community’s wellbeing as a very important or somewhat important reason to get tested for COVID-19.

### Reassurance of negative status

Participants described their feelings of reassurance from having access to routine COVID-19 diagnostic testing because testing could inform them of their negative status. Staff in particular said that testing negative every week mitigated their fears of exposure to COVID-19 in the workplace and allowed them to work at school more comfortably.

Workplaces were a site of specific concern: From the survey, 63.3%(n = 88) of staff believed they were likely to get infected with COVID-19 compared to only 40.6% of parents (n = 54). School staff members in our FGDs repeatedly discussed their fears of being at heightened risk of exposure due to their proximity to their students in crowded classroom spaces. Typical risk mitigation strategies, such as consistent mask wearing, physical distancing, symptom checking, and hand washing were difficult to enforce at times. Negative results that staff received from voluntary routine testing reassured them that they were safe, they could continue to work, and they were not transmitting the virus to their students or their loved ones at home.

I personally feel safer at school with it to be honest with you. I feel like going into the classroom with 26 kids...a classroom full of kids where there’s no way to social distance was a little bit daunting. I was a little nervous about doing it without dividers, without you know, pretty much anything and so when the results started coming in...that makes me feel comfortable. That makes me feel a lot better about it. -School Staff

## Discussion

Our findings describe the ways in which testing has discernable costs for socially vulnerable school communities, and how the burden of testing can be structural and largely outside the bounds of individual control. The COVID-19 pandemic has called attention to the glaring inequities in testing uptake for low-income, minority, and non-English speaking families in particular [18, 23]. Although individual hesitancy towards the test itself or distrust of broader health systems may be significant drivers of lower testing uptake in more socially vulnerable communities, it is important to consider the broader logistical challenges that have interfered with community members’ intentions and abilities to engage in testing behaviors.

When implementing COVID-19 testing as a school risk mitigation strategy, we must consider differing attitudes towards testing to reconcile the tension between the consequences of a positive diagnosis and the potential reassurance brought about by a negative diagnosis. Our findings have been consistent with other studies that have recognized this conflict between school members feeling safer at school as a result of testing while simultaneously expressing their concerns about the burdens of testing, such as testing stigma and quarantine and isolation requirements [11, 24]. While there was a broad consensus among our participants regarding the utility of testing in keeping schools open, of note are the concerns school members expressed regarding the disruption of a positive test result in their own lives and the lives of other families within their school communities. Schools may be able to address some of this apprehension to encourage more of their community members to test. From our participant’s views, this includes providing testing materials and isolation and quarantine resources in multiple languages to families to make this information more accessible, discussing COVID-19 related stigma at community meetings and school assemblies, and framing testing as a family and community benefit rather than just an individual one. Similarly, a review of the literature [25] found that adapting testing to the needs of the community through improved accessibility and increased awareness is essential in overcoming barriers. However, schools alone do not have the capacity to address the more daunting structural barriers to testing that were highlighted by our study participants. Our own County funded SASEA project provided free testing and accompanying medical staff to our partnered schools and shows how broader structural support was vital in these operations. Similar community-oriented testing programs supported from the state to federal level like Say Yes! Covid Test Michigan Program [26] or the promotora-centered COVID-19: Healthy Oregon (Oregon Saludable) [27] show similar success in increasing testing uptake among socially-vulnerable populations and reduced cases of COVID-19 within the community. Collaborations of these types of programs with schools would foster a supportive testing culture in locations that feel comfortable and accessible to students, parents, and staff.

Our findings have also called attention to how testing decisions in socially vulnerable communities are often undergirded by considerations of personal financial circumstances, as many families simply cannot afford to test positive. Multiple studies have cited income or job loss as a key factor for COVID-19 testing hesitancy [25, 28–31]. Additionally, individuals who have lower educational attainment, poorer health outcomes, and lower socioeconomic status are also significantly more likely to be employed in forms of essential or precarious work that places them at higher risk of COVID-19 exposure [13]. This illustrates a vicious cycle where those of lower socioeconomic status are at greatest risk of COVID-19 exposure through their work, but must isolate or quarantine to the detriment of their already precarious financial situation. Given the overwhelming structural barriers cited by our participants, these communities need to feel empowered in their decisions to get tested for COVID-19 without feeling that their or their family’s livelihoods are being threatened. Additionally, if families feel that they have the social support to isolate or quarantine successfully through paid sick leave, food assistance, rental assistance, and eviction moratoriums, they may feel less hesitant about COVID-19 testing [31].

It is important to note that school community members also cited multiple facilitators to testing — protecting family health, protecting community health, and being reassured by a negative status — that could be leveraged to motivate others within the school community to test. While much of the emerging literature often focuses on barriers and strategies to overcome barriers to COVID-19 testing, these findings provide novel insights on how school community members can be motivated to test. Our findings underscore how testing may be perceived by school community members as a punitive measure with far-reaching social and financial consequences for individuals who receive a positive diagnosis. While it is important for community members to be provided with the necessary support and resources to manage these social and financial consequences, the individual, interpersonal, and community-wide benefits of testing should be continuously communicated. Even with the protections afforded by the COVID-19 vaccine, diagnostic testing remains a critical strategy to halt further transmission and prevent future outbreaks.

## Conclusion

Testing is a critical risk mitigation tool that has been integral in maintaining the safe operation of schools, and will continue to be necessary so long as the risk of exposure to COVID-19 remains inherent in public spaces. Schools must continually communicate the necessity of testing to their community members. Public health efforts must also address the pressing structural barriers to testing while taking into consideration the unique concerns that socially vulnerable communities have. These efforts must recognize how their abilities or desires to seek out testing are inhibited by factors beyond their control. As the pandemic is dynamic, current and future disease prevention efforts must continue to incorporate testing as a strategy to maintain school safety, facilitate access for vulnerable community members, and invest in the implementation of robust testing systems to prevent reinforcing the vulnerabilities of the most disadvantaged populations.

## Data Availability

The datasets generated and/or analyzed during the current study are not publicly available due to the confidential nature of focus group discussions but are available from the corresponding author on reasonable request.

## List of Abbreviations

CDC: Centers for Disease Control and Prevention
FGD: Focus Group Discussions
SASEA: Safer at School Early Alert
SVI: Social Vulnerability Index
